# Burst fracture of the lumbar vertebra due to a landmine injury: a case report

**DOI:** 10.4076/1757-1626-2-6257

**Published:** 2009-06-24

**Authors:** Serkan Bilgic, Volkan Kilincoglu, Mustafa Kurklu, Yuksel Yurttas, Huseyin Ozkan, Ali Sehirlioglu

**Affiliations:** Department of Orthopaedics, Gulhane Military Medical AcademyEtlik, AnkaraTurkey

## Abstract

**Introduction:**

The reason we report this case is that spine injuries may well occur due to landmines similar to other injuries like traumatic limb amputations and more over they may be overlooked.

**Case presentation:**

The patient was 29-years-old Turkish male and was a member of the military. He detonated the landmine that caused his injuries while in a conflict zone. He had a right below knee and left above knee traumatic amputations. He had also mild intermittent pain in his lower back. There were no focal neurological findings such as weakness, altered sensibility, or alteration in the function of the bowel or bladder. Radiographs of the lumbar spine revealed an L2 burst fracture. Computed tomography scans and magnetic resonance imaging of the lumbar spine demonstrated a burst fracture of the L2 vertebrae and moderate compression in the anterior portion of the thecal sac due to the fracture fragment. Because of the stabile nature of the L2 burst fracture and lack of neurological disturbance, operative decompression, instrumentation and fusion was not performed. After healing of the stumps, the patient was mobilized with immediate prostheses and a thoracolumbosacral brace.

**Conclusion:**

Spine injuries should not be overlooked when evaluating patients after landmine explosions. After the patient has been stabilized, the secondary screening and radiographic evaluations should also comprise the thoracic, thoracolumbar and lumbar spine when treating patients after landmine injuries.

## Introduction

Landmines have become widely used in military conflicts since the end of World War II. Their popularity is the result of several factors including the simplicity of their production, their low cost and ease of distribution in the field. Now there are at least 50 million landmines buried in 71 countries throughout the world, and 2-5 million new landmines are planted every year [1-3].

Despite active landmine education programs and de-mining efforts in many nations [4] tens of thousands of people are killed or injured by these weapons every year [5]. The type of device and the part of the body coming into contact with the landmine at the time of detonation determine the injury pattern. The blast and thermal effects of the landmine cause injuries that usually involve the lower limbs and the perineum [6-9]. The spine may also be injured with blast mines but there is no detailed knowledge related to this topic in english literature.

The purpose of this case is to report that a spine injury may be occured with the other injuries such as traumatic limb amputations due to a landmine and it may be overlooked.

## Case presentation

The record of the patient who sustained a spine injury with bilateral limb amputations due to a landmine explosion in Turkey was reviewed. He was 29-years-old Turkish male and military personnel. He had stood on the pressure plate and detonated the landmine in a conflict zone. He had been initially evaluated by military general practitioner at the point of injury and had been transferred to regional military hospital following first aid. He had been hospitalized in there because of his multipl injuries for four days. Thereafter he had been transferred to intensive care unit of our hospital.

**Figure 1.**
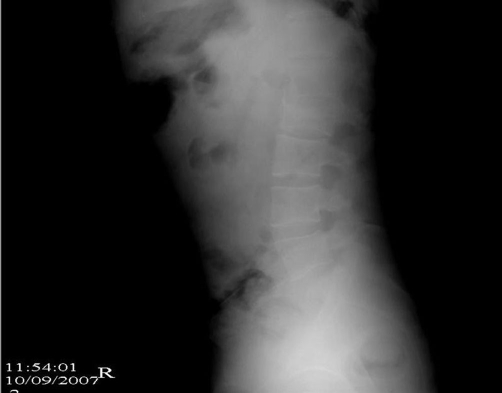
Lateral X-Ray of the case.

**Figure 2.**
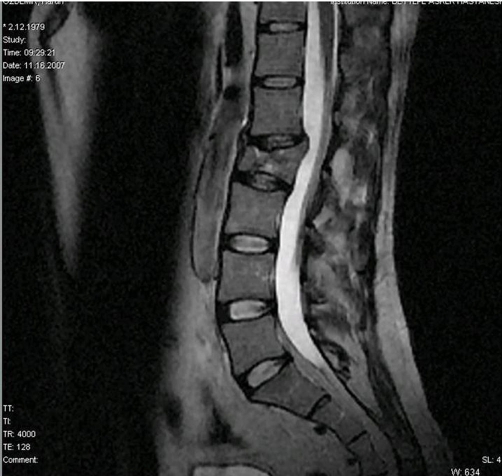
Sagittal MRI imaging shows mild compression of the spine cord.

**Figure 3.**
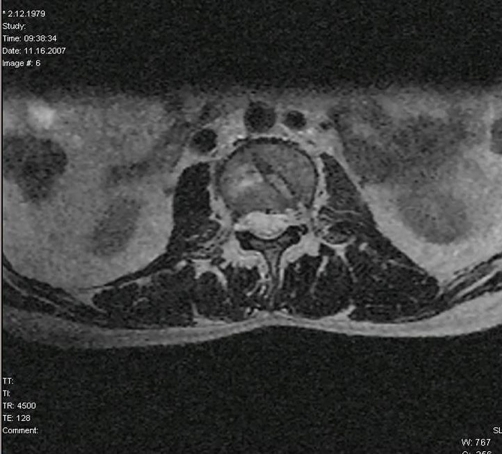
Axial MRI imaging shows mild compression of the spine cord.

All the physical and radiological examinations were done at the time of admission. There was no pathological findings in abdominal, head and thoracic parts of the body. He had right below knee and left above knee traumatic amputation, multiple skin lacerations which contain foreign bodies and left forearm large skin injury. He had also mild intermittent pain in his lower back. The posterior paralumbar muscles were tender. He did not have any neurological findings such as an alteration in the function of the bowel or bladder. Radiographs of the lumbar spine revealed L2 burst fracture (Figure 1). Afterward CT scans and MRI were performed to evaluate spinal cord, bone and ligamentous injury. CT scans and MRI of the lumbar spine demonstrated burst fracture of the L2 and mild compression in the anterior portion of the spinal canal due to the fracture fragment (Figure 2 and 3).

Stumps of both amputees which were necrotic and infected treated with left hip disarticulation and right knee disarticulation. Left forearm skin injury was treated with skin flap. Primary closure of wounds, debridement of necrotic tissues, removal of the foreign bodies were also performed with revision of the traumatic amputations.

Because of the stabil nature of the L2 burst fracture and lack of neurological disturbance operative decompression, enstrumentation and fusion was not performed. After healing of the stumps the patient was mobilized with immediate prostheses and TLSO brace.

## Discussion

A patient who had L2 burst fracture with bilateral limb amputations due to landmine explosion was treated and his data was reviewed. Anti-personnel landmines, a type of weapon designed to disable or kill, have been used in large numbers [8] in almost every conflict since the Second World War.

Blast mines are usually buried and are detonated by foot pressure. The detonation of a blast mine produces a transient pressure wave and an overpressure of hundreds of pounds per square inch. The blast and thermal effects of the landmine cause injuries that usually involve the lower limbs, the perineum and the upper half of the body. L2 burst fracture in our patient must have been with this mechanism and this injury was fall after the detonation. Other type of injury patterns are caused by fragmentation devices producing widespread low-velocity missile injuries to head, chest, abdomen and major blood vessels and injuries to face and/ or hands as a result of a landmine exploding at a close range - usually in a landmine - clearer or a child.

The blast and thermal effects of the landmine usually result in traumatic amputation of the leg, either above or below the knee [7]. Soft-tissue damage to the contralateral limb may also become related to the size of the explosive charge in the mine. Large mines may also injure the perineum [10].

The spine is rarely injured. But if spine injury occurs it may cause devastating clinical course and results. So entire spinal column should be assessed properly in landmine injuries especially in the consciousness patients. In our patient the complaint related to spine was appeared at admission. Therefore we determined the L2 burst fracture quite early. The severity of traumatic amputations may conceal the other serious conditions initially.

In conclusion; spine injuries which have a relatively high morbidity, a high requirement for spinal surgery and possible high neurological risk should be reminded in landmine explosions. After plain cervical, pelvic, chest radiographs, and stabilization of the patient the proper radiographs including thoracolumbar radiographs should be obtained in landmine injuries.

## List of abbreviations

CT: Computed tomography
MRI: Magnetic resonance imaging
TSLO: Thoracolumbosacral orthosis
